# Caffeine Intake, Short Bouts of Physical Activity, and Energy Expenditure: A Double-Blind Randomized Crossover Trial

**DOI:** 10.1371/journal.pone.0068936

**Published:** 2013-07-15

**Authors:** Pedro B. Júdice, Catarina N. Matias, Diana A. Santos, João P. Magalhães, Marc T. Hamilton, Luís B. Sardinha, Analiza M. Silva

**Affiliations:** 1 Exercise and Health Laboratory, Interdisciplinary Center for the Study of Human Performance, Faculty of Human Kinetics, Technical University of Lisbon, Cruz-Quebrada, Portugal; 2 Inactivity Physiology Laboratory, Pennington Biomedical Research Center, Baton Rouge, Louisiana, United States of America; Universidad Europea de Madrid, Spain

## Abstract

PA energy expenditure (PAEE) is the most variable component of Total Energy Expenditure (TEE) and largely due to the balance of sedentary time (SedT) and low intensity physical activity (LIPA). There has been an emergence for seeking an understanding of factors which determine variations in SedT, LIPA, and PAEE. Sedentary behavior and physical activity are relatively resistant to change by experimental dietary treatments and significant body weight changes. Although caffeine (Caf) is by far the most heavily used nutritional agent ingested to promote a sense of vigor/alertness, it is still unknown if Caf is effective in increasing PAEE and physical activity. The aim of the study was to test the hypothesis that 2 daily doses of Caf (as a capsule to blind the treatment and divided equally during breakfast and lunch) increase PAEE and TEE, and it would do so through increasing the frequent and brief bouts of physical activity (~1-5 min long) through the day as measured by accelerometry. In 21 low Caf users (<100 mg day^-1^), we used a double-blind crossover trial (ClinicalTrials.govID;NCT01477294) with two conditions (4-day each with a 3-day washout period) randomly ordered as 5 mg kg^-1^ day^-1^ of Caf and maltodextrin as placebo (Plc). Resting energy expenditure (REE) by indirect calorimetry, total energy expenditure (TEE) from doubly labeled water, PAEE calculated as TEE-(REE+0.1TEE), and accelerometry measurements of both LIPA and MVPA were not different between conditions. However, regardless of caffeine or placebo, there were several significant relationships between brief bouts of LIPA and MVPA with PAEE. In conclusion, this double-blind study found that low and moderate-vigorous activity as well as the total volume of PAEE in free-living conditions is resistant to dietary caffeine intake that was equivalent to 5 cups of espresso or 7 cups of tea.

**Trial Registration:**

ClinicalTrials.gov NCT01477294

## Introduction

Caffeine is one of the most widely consumed food ingredients, commonly found in beverages including coffee, tea and soft drinks, as well as in products containing cocoa or chocolate, and a variety of medications and dietary supplements [1,2]. It has been considered as a thermogenic agent [3–5] that could help in preventing a positive energy balance and obesity [6,7]. Although the effect of caffeine on energy expenditure (EE) has been the subject of numerous investigations [8–10], its effects on daily free living physical activity energy expenditure (PAEE) and physical activity patterns, specifically the frequency of short bouts of low intensity physical activity (LIPA) or moderate to vigorous physical activity (MVPA) have not hitherto been studied.

Current PA guidelines for adults are focused on increasing MVPA levels. However, recent data indicate that only about 4-6% of the Portuguese adult population meet these recommendations [11] which is in accordance with the results from the US adult population (5%) [12]. A recent operational definition [13] defined sedentary behavior as any waking behavior characterized by an energy expenditure ≤1.5 METs while in a sitting or reclining posture. Sedentary behavior may be one of the causes of many modern day chronic diseases including insulin resistance, type 2 diabetes and metabolic syndrome [14–16], and occupation-related sedentary behavior may account for a significant portion of the increase in mean body weight for women and men [17]. Recommending the accumulation of enough time in multiple short bouts of PA, at a low intensity (i.e., LIPA) distributed throughout the day may be an effective alternative for the reduction of sedentary behavior [18]. It was hypothesized by Hamilton et al. in 2007 [14] “that any type of brief, yet frequent, muscular contractions throughout the day may be necessary to short-circuit unhealthy molecular signals causing metabolic diseases”. Experimental [19] and observational [20–22] studies have only begun to suggest indirectly that interrupting sedentary behavior with short bouts of LIPA or MVPA may be positively associated with relevant health outcomes such as blood lipids, glycemic levels, insulin resistance and systolic blood pressure. However, changing human behavior to become more physically active in free-living conditions is very difficult to do, and the simple fact that caffeine is so widely used in all cultures is intriguing to consider if it provides enough of a stimulus to effect a more active behavioral change. Nevertheless, research studies are still lacking regarding the effective contribution of short bouts frequency on PAEE assessed by double labeled water under free-living conditions. In fact, only one study has investigated the relationship between PA short bouts lasting less than 10-min with EE [23] in laboratorial conditions using indirect calorimetry.

Taken together, the absence of published studies concerning the role of caffeine on PA patterns and the contribution of short bouts frequency of LIPA or MVPA to free-living PAEE led us to conduct this investigation with two purposes: a) To evaluate the impact of a moderate dose of caffeine during a 4-day period on PAEE and daily number of short bouts of at least 1 up to 5-min of either LIPA or MVPA in non-obese, physically active males under free-living conditions; b) to analyze, either under caffeine or placebo treatments, the associations between short bouts lasting 1 up to 5-min of LIPA and MVPA with PAEE assessed from doubly labeled water in combination with indirect calorimetry.

## Materials and Methods

### Ethics Statement and Participants

This research has been approved by the ethics Committee of the Faculty of Human Kinetics, Technical University of Lisbon and were conducted in accordance with the declaration of Helsinki for human studies [24]. All participants were informed about the possible risks of the investigation before giving their written informed consent to participate.

**Figure 1:**
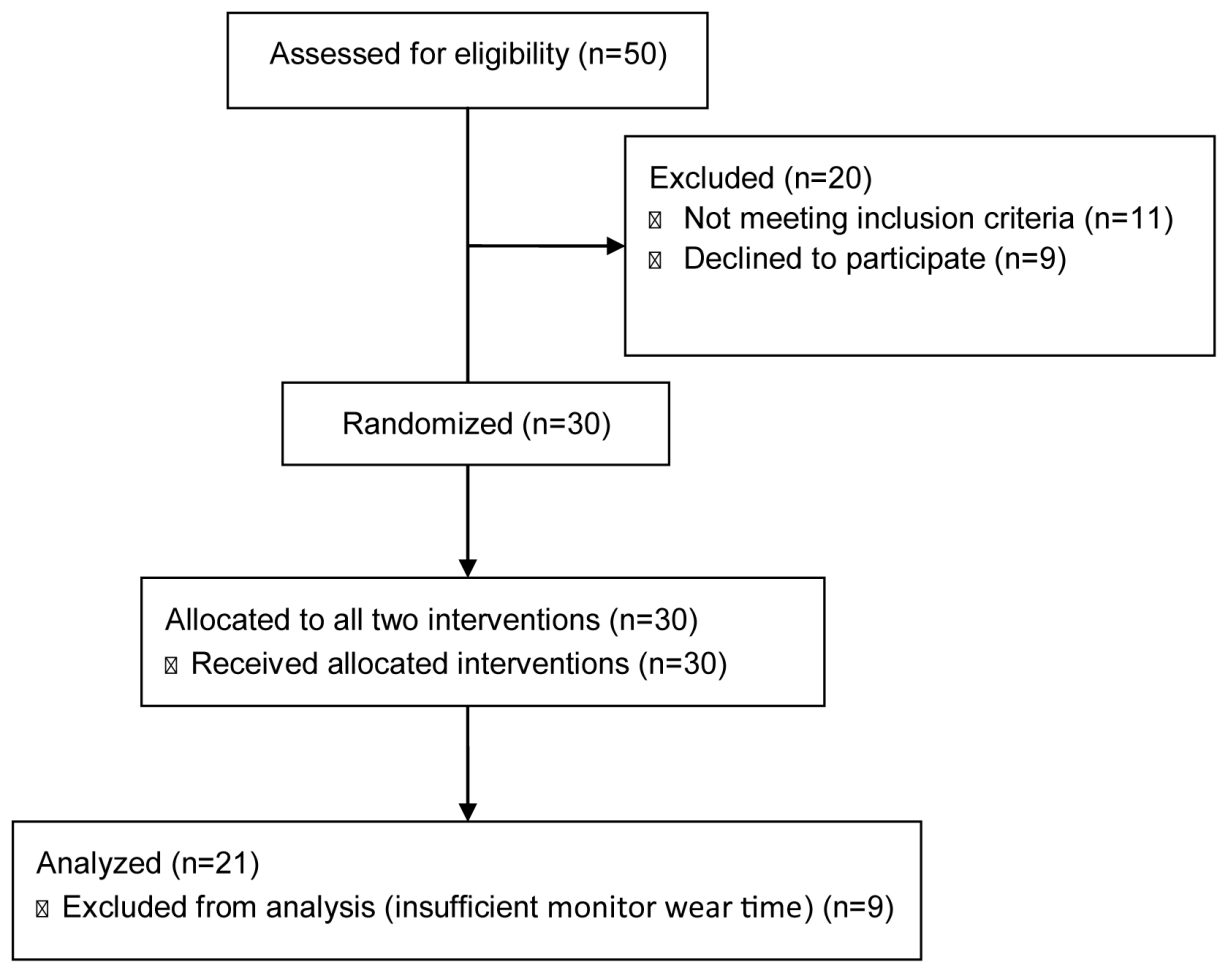
Screening, enrollment and interventions of the study participants.

A total of 30 healthy nonsmoking males were considered for eligibility according to a screening algorithm (Figure 1). Participants were recruited through advertisements placed nearby the institution and volunteered to participate in this study. Inclusion criteria were: age between 20 and 39 years old; body mass index (BMI) between 18.5 and 29.9 kg m^-2^; not taking any medication or dietary supplements that may affect energy expenditure; physically active (estimated by IPAQ), which represent the accumulation of at least 30-min per day of moderate-to-vigorous physical activity, according to the recommendations of the World Health Organization [25]; and low-caffeine users (<100 mg day^-1^) [26]. The daily consumption of caffeine was estimated based on 7-day self-report of the daily intakes of coffee, tea, caffeinated sodas, chocolate, and other dietary sources, according to a list provided by two sources [27,28].

### Experimental design

Participants were followed in a double-blind crossover experimental design (ClinicalTrials.govID;NCT01477294) with two conditions in a random sequence (a detailed description of the methods are available as supporting information; Protocol S1, CONSORT checklist S1). Caffeine/placebo conditions were randomized by an automated computer-generated randomization scheme and assigned to specific study days. The participants as well as the study personnel, who delivered the capsules to the participants and performed all tests, were blinded to the condition allocation. A laboratorial technician, responsible for preparation of the doses, was the only person aware of the randomization code during the trial. She was not involved in other study functions. Caffeine (5 mg per kg of body mass per day) and maltodextrin as placebo, both through capsules and divided equally during breakfast and lunch. Each condition lasted for 4 days and participants were instructed to keep the same eating patterns and level of PA. There was a washout period of 3 days between each condition [29]. Moreover, to reduce the variability of individual PA patterns during the week, both conditions were performed on the same weekdays while the washout period always included the weekend days. Evaluations were performed at three time points: 1) Baseline: First visit for collecting the initial measurements; 2) Condition 1: second visit, 4 days after baseline, for collecting the final measurements of the first randomly assigned condition (placebo or caffeine); and 3) Condition 2: third visit, 7 days after the end of the first condition, including the 3-day washout period, for collecting the final measurements of the second randomly assigned condition (placebo or caffeine).

Participants were required to fast for at least 12 h prior to each visit, refrain from vigorous exercise for at least 15 h, alcohol consumption for 24 h, and consume a normal evening meal the night before the visit. All measurements were carried out in the same morning of the same week day. In brief, the procedures adopted were as follows:

### Caffeine and placebo intake

After weighing the participants, the dose was individually prepared to assure that a 5 mg of caffeine per kg of body mass per day was administered. The dose of caffeine was divided into two equal parts (2.5 mg kg^-1^) to be orally consumed through capsules in the morning and after lunch. An equivalent dose (5 m kg^-1^day^-1^) and number of placebo capsules, of the same color as the caffeine capsules, containing maltodextrin were provided for the placebo condition.

### Body composition measures

#### Anthropometry

Participants wearing minimal clothes and without shoes were weighed to the nearest 0.01 kg, on an electronic scale connected to a plethysmograph computer (BOD POD^®^, COSMED, Rome, Italy). Height was measured to the nearest 0.1 cm with a stadiometer (Seca, Hamburg, Germany) according to the standardized procedures described elsewhere [30]. BMI was calculated as body mass (kg) height^-2^ (m).

#### Fat mass (FM) and fat free mass (FFM)

Dual energy X-ray absorptiometry (DXA) (Hologic Explorer-W, fan-beam densitometer, software QDR for windows version 12.4, Waltham, Massachusetts, USA) was used to estimate FM and FFM. The equipment measures the attenuation of X-rays pulsed between 70 and 140 kV synchronously with the line frequency for each pixel of the scanned image. Following the protocol for DXA described by the manufacturer, a step phantom with six fields of acrylic and aluminium of varying thickness and known absorptive properties was scanned to serve as an external standard for the analysis of different tissue components. The same technician positioned the participants, performed the scans and executed the analysis according to the operator’s manual using the standard analysis protocol.

### Resting energy expenditure

Resting energy expenditure (REE) was assessed in the morning (7.00–11.00 a.m.), 1-hour after the first half-dose of caffeine (2.5 mg kg^-1^) or placebo to be orally consumed through capsules. All measurements were performed in the same room at an environmental temperature and humidity of approximately 22^°^C and 40-50%, respectively.

The MedGraphics CPX Ultima (Medical Graphics Corp, St Paul, MN, with Breeze suite software) indirect calorimeter was used to measure breath-by-breath oxygen consumption (*VV*O_2_) and carbon dioxide production (*V*CO_2_) using a facial mask. One trained technician conducted all measurements. The oxygen and carbon dioxide analysers were calibrated in the morning before testing using known gas concentration. The flow and volume were measured using a pneumotachograph calibrated with a 3 L-syringe (Hans Rudolph, inc. TM). Device auto calibration was performed between participants. Before testing, participants were instructed about all the procedure and asked to relax, breathe normally, not to sleep, and not to talk during the evaluation. Total rest duration was 60-min, participants lied supine for 30-min covered with a blanket and the calorimeter device was then attached to the mask and breath by breath *V*O_2_ and *V*CO_2_ were measured for another 30-min period. Outputs of *V*O_2_, *V*CO_2_, respiratory exchange ratio (RQ), and ventilation were collected and averaged over 1-min intervals for data analysis. The first and the last 5-min of data collection were discarded and the mean of a 5-min steady state interval between the 5 and the 25-min with RQ between 0.7 and 1.0 was used to determine REE. Steady state was defined as a 5-min period with ≤ 10% CV for *V*O_2_ and *V*CO_2_ [31]. The mean *V*O_2_ and *V*CO_2_ of 5-min steady states were used in Weir equation [32] and the period with the lowest REE was considered.

### Total and physical activity energy expenditure

Total energy expenditure (TEE) was measured by doubly labeled water (DLW) [33,34]. Deuterium oxide and 18-Oxygen were administered in the morning of the first visit (baseline). Briefly, participants were weighed in the morning and baseline urine was collected. An oral dose of 2.7 g kg^-1^ of TBW of a 10 atom% (AP) solution of H_2_^18^O (Taiyo Nippon Sanso Corporation, Tokyo, Japan), assuming that TBW is 0.61xTotal body weight, and 0.24 g kg^-1^ of TBW of a 99.9 AP solution of 2H_2_O (Sigma-Aldrich, Co, St Louis, Mo, USA), diluted in 50 ml of water and administered to the participants at 7.00 a.m. Post-dose urine samples of the first visit day were taken and stored from voids at 4 and 5h. Morning urine samples and 1h after were collected on day 4 (end of first condition), after the washout period (day 7), and at the end of the second condition (last day). Urine samples were prepared and filled with the equilibration gas. The equilibration period lasted for 3 days and 8h, respectively for ^2^H and ^18^O. Samples were analyzed in duplicates and calibrated against standard mean ocean water (SMOW), using Hydra isotope ratio mass spectrometer (PDZ, Europa Scientific, UK). A two-point sample method was used to evaluate the elimination constants (kd and ko, respectively for deuterium and 18-oxygen) over the first and the second 4-day periods (condition 1 and 2, respectively). A similar procedure was used elsewhere [35]. For analyzing condition 2, urine samples collected after the washout period (day 7) and on the last day of the trial were considered to evaluate the elimination constants. Energy expenditure measured by the DLW method was calculated from a modified Weir’s equation by use from DLW and calculated from the food quotient obtained by dietary intake records [32].

Physical activity energy expenditure (PAEE) was calculated as the difference between TEE and the sum of REE with 0.1*TEE (assuming the thermic effect of food is ~10% of TEE).

### Physical activity patterns and short bouts assessment

All participants were asked to wear an accelerometer (*ActiGraph, GT1M model, Fort Walton Beach, Florida*) on the right hip, near the iliac crest during eleven consecutive days, including two weekend days [36]. The delivery and reception of the accelerometers to the participants, as well the explanation of its use, were made personally [37]. The devices were activated on the first day at 7.00 a.m. and data were recorded in 10-sec epochs. The device activation and data download were performed using the software Actilife Lifestyle (v.3.2). Processing was performed using the software MAHUffe v.1.9.0.3 (available at www.mrc-epid.cam.ac.uk) from the original downloaded files (*. dat). For the analyses, a valid day was defined as having 600 or more min (≥10 hours) of monitor wear, corresponding to the minimum daily use of the accelerometer [37]. Apart from accelerometer non-wear time (i.e. when it was removed for sleeping or water activities), periods of at least 60 consecutive min of zero activity intensity counts were also considered as non-wear time.

For the short bouts frequencies assessment, we analyzed the number of bouts of at least 1 up to 5-min individually and for both PA of at least low and of at least moderate intensity, using the MAHUffe software. For assessing the frequency of short bouts of LIPA, we calculate the difference between the number of bouts of at least low from those of moderate intensity PA, considering the bouts durations defined (1 up to 5-min).

The amount of activity assessed by accelerometry was expressed as the min per day spent in different intensities and the frequency of short bouts of at least 1 up to 5-min of low (LIPA) and at least moderate (MVPA) intensity PA. The cutoff values used to define the intensity of PA and therefore to quantify the mean time in each intensity (sedentary, low, moderate or vigorous) were: sedentary: < 100 counts min^-1^; low: 100-2019 counts min^-1^; moderate: 2020-5998 counts min^-1^ (corresponding to 3-5.9 METs); vigorous: ≥ 5999 counts min^-1^ (corresponding to ≥ 6 METs) [12].

### Energy and nutrient intake

Food intake was assessed throughout the study using 24-h diet records during the 11-day period of this trial. Participants were instructed regarding portion sizes, food preparation aspects, and others aspects pertaining to an accurate recording of their energy intake. Accuracy of the food intake recordings was ascertained by the study nutritionist at the second study visit (4 days after baseline). At the last visit, records were turned in and reviewed for water ingestion, macronutrient composition and total energy intake by the same nutritionist. Diet records were analyzed using a software package (Food Processor SQL).

### Statistical analysis

Statistical analysis was performed using PASW Statistics for Windows version 18.0, 2010 (SPSS Inc., an IBM Company, Chicago IL, USA). Descriptive analysis included means ± SD for all measured variables. Normality was explored using the Shapiro-Wilk test.

Comparison of means between conditions was performed using paired sample T-Test. To compare the effects of caffeine on mean values of the main dependent variables, linear mixed models for repeated measures were used.

In addition, to evaluate the effect of caffeine on the dependent variables, fat-free mass and energy intake were used as covariates.

To evaluate the association between short bouts of at least 1 up to 5-min of either LIPA or MVPA with PAEE, multiple regression analysis was performed, adjusting for sedentary time. Unstandardized residuals were explored to check homoscedasticity and outliers.

Reliability for all the methods employed in this study was assessed using the coefficient of variation (CV). Based on test–retest using ten participants, the coefficient of variation (CV) in our laboratory for FM and FFM are 1.7% and 0.8%, respectively [38], 4.3% for TEE, and using seven participants CV for REE is 4.0%.

Statistical significance was set at *p* < 0.05.

### Power Calculation

Considering EE, prior data indicate that the difference in the response of matched pairs is normally distributed with standard deviation of 120 Kcal [39]. Considering a true difference of 77 Kcal [39] in the mean response of matched pairs, 21 pairs of participants are necessary to reject the null hypothesis that the response difference is zero with a power of 80% and a type I error probability of 0.05.

Regarding the larger variability in EE values due to the free living settings we enrolled 30 participants.

## Results

Twenty one participants were included in the study (Figure 1). The participant’s characteristics and body composition at baseline are presented in Table **1**.

**Table 1:** Participant’s characteristics and body composition (*N*=21).

	**Mean ± SD**	**Range**
**Age (years)**	24.3 ± 4.5	20–38
**Height (m)**	1.76 ± 0.07	1.64-1.87
**Body mass (kg)**	72.4 ± 9.4	51.8-90.2
**BMI (kg m^-2^)**	23.7 ± 2.4	19.6-27.7
**FM (kg)**	11.7 ± 4.3	5.1-20.6
**FFM (kg)**	59.9 ± 6.9	46.2-70.3

Abbreviations: SD, standard deviation; N, number of participants; BMI, body mass index; FM, fat

mass; FFM, fat-free mass.

The different components of energy expenditure, physical activity and dietary patterns, during treatment conditions are presented in Table **2**.

**Table 2:** Total, resting, and physical activity energy expenditure, habitual physical activity, and dietary intake under treatment conditions (*N* = 21).

	**Placebo**	**Caffeine**
	Mean ± SD	Mean ± SD
**TEE (kcal day^-1^)**	3107 ± 438	3046 ± 542
**REE (kcal day^-1^)**	1442 ± 240	1428 ± 258
**PAEE (kcal day^-1^)**	1354 ± 373	1368 ± 359
**Daily Steps**	8527 ± 2910	9374 ± 3158
**Sedentary (min day^-1^)**	703 ± 87	709 ± 94
**Low (min day^-1^)**	151 ± 38	157 ± 38
**Moderate (min day^-1^)**	45 ± 21	49 ± 24
**Vigorous (min day^-1^)**	5 ± 6	6 ± 5
**Energy Intake (kcal day^-1^)**	2682 ± 641	2472 ± 391
**Carbohydrates (g)**	309 ± 86	298 ± 56
**Fat (g)**	92 ± 24	79 ± 21
**Protein (g)**	124 ± 35	115 ± 21

Abbreviations: SD, standard deviation; N, number of participants; TEE, total energy expenditure; REE, resting energy expenditure; PAEE, physical activity energy expenditure; Min, minutes

No differences were observed between treatment conditions for PAEE (-13.7 ± 248.0 kcal day^-1^; p=0.803), TEE (60.8 ± 417.4 kcal day^-1^; p=0.512), REE (13.6 ± 153.1 kcal day^-1^; p=0.689), steps (-847 ± 3352 steps day^-1^; p=0.261), and time spent in sedentary behavior (-7 ± 108 min; p=0.783), low (-6 ± 30 min; p=0.394), moderate (-3 ± 25 min; p=0.549), and vigorous (-1 ± 6 min; p=0.430) intensities. After controlling for fat free mass and energy intake, the results remained the same (p>0.05) for all the aforementioned variables. Also, dietary intake did not differ between caffeine and placebo treatment conditions, specifically with respect to energy intake (p=0.105) and the amount of carbohydrates (p=0.553), fat (p=0.052), and protein (p=0.225) consumed. Therefore, the food quotients were not different in both conditions, which reduced the variability within the DLW method.

Table **3** shows the frequency of PA divided by the intensity and the bout duration for both conditions.

**Table 3:** The daily physical activity levels by bout duration on both conditions (*N* = 21).

Bout duration	**Placebo**		**Caffeine**	
	Frequency (bouts day^-1^)			
	**LIPA**	**MVPA**	**LIPA**	**MVPA**
**> 1 min**	93 ± 44	35 ± 22	99 ± 42	36 ± 21
**> 2 min**	35 ± 19	19 ± 15	39 ± 23	19 ± 12
**> 3 min**	15 ± 9	10 ± 9	17 ± 10	10 ± 7
**> 4 min**	9 ± 7	7 ± 7	10 ± 7	6 ± 5
**> 5 min**	7 ± 6	6 ± 6	7 ± 4	5 ± 4

The data are expressed as the means with standard deviations (mean ± SD)

Abbreviations: LIPA, low physical activity; MVPA, moderate-to-vigorous physical activity; Min, minutes

The results showed no significant differences (*p* ≥ 0.05) between placebo and caffeine conditions with mean differences for the frequencies of short bouts of at least 1 (-11 ± 41), 2 (-7 ± 20), 3 (-3 ± 10), 4 (-3 ± 7), and 5 (-1 ± 5) min of LIPA. Similarly, there were no significant differences between conditions, for the MVPA short bouts of at least 1 (-6 ± 23), 2 (-2 ± 14), 3 (-1 ± 8), 4 (0 ± 6), and 5 (0 ± 5) min. After controlling for fat free mass and energy intake, the results remained the same (p>0.05).

**Figure 2:**
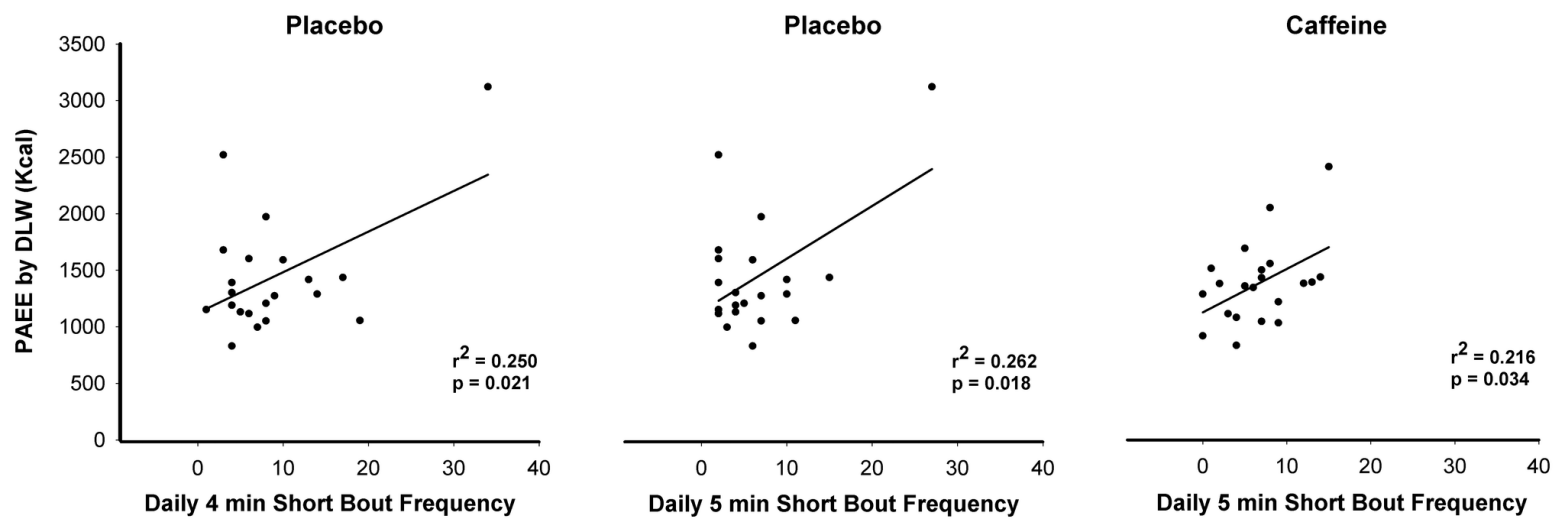
Physical activity energy expenditure and frequency of low intensity short bouts (>4-min), under both conditions. Association between physical activity energy expenditure (PAEE) from doubly labeled water (DLW) and the frequency of short bouts (>4 min) performed at a low intensity physical activity, under placebo and caffeine conditions.

Concerning the second purpose of this investigation, to analyze the association between short bouts and PAEE under caffeine and placebo, Figure **2** shows the relationship between the daily frequencies of LIPA by the bout duration with PAEE, for both placebo and caffeine conditions.

As illustrated in Figure **2**, for LIPA, short bouts lasting longer than 4 (9 ± 7) and 5 (6 ± 6) min under placebo, and at least 5 (7 ± 4) min under caffeine predicted PAEE [placebo: β=36, p=0.021 (4-min); β=47, p=0.018 (5-min); caffeine: β=38, p=0.034 (5-min), respectively]. Therefore, our results demonstrated an additional 36 kcal bout^-1^, and 38 kcal bout^-1^ in PAEE for each bout of at least 4-min, under placebo and caffeine respectively. These associations remained significant when adjusted for sedentary time (<100 cpm).

**Figure 3:**
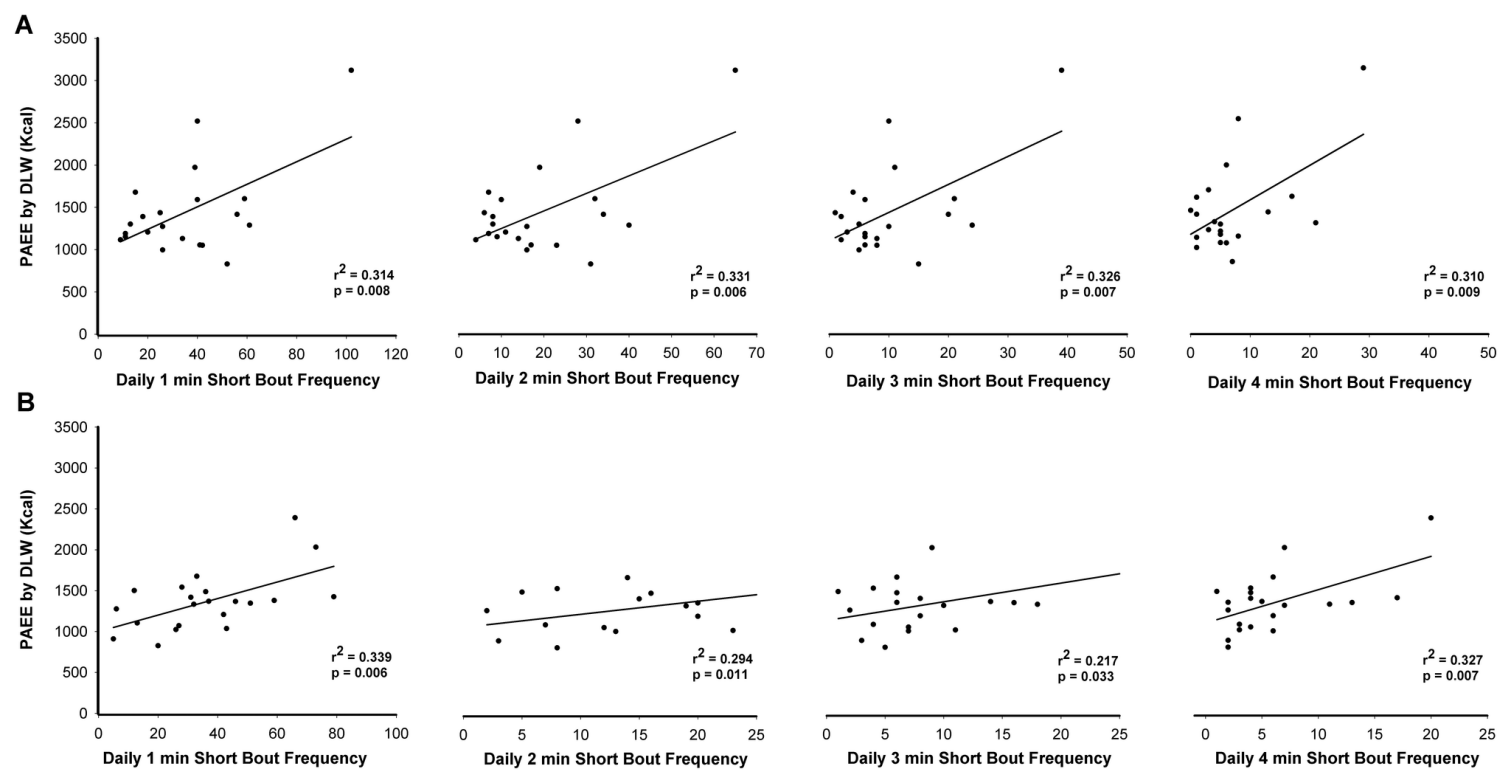
Physical activity energy expenditure and frequency of moderate-to-vigorous intensity short bouts, under placebo-(A) and caffeine-(B). Association between physical activity energy expenditure (PAEE) from doubly labeled water (DLW) and the frequency of short bouts (1-4 min) performed at a moderate-to-vigorous physical activity intensity, under placebo (panels A) and caffeine (panels B).

The associations between the frequencies of MVPA short bouts with PAEE are presented in Figure **3**, under placebo (A) and caffeine (B) treatment conditions, respectively.

MVPA short bouts lasting longer than 1 (34 ± 23), 2 (19 ± 15), 3 (10 ± 9), and 4 (7 ± 7) min under placebo condition, and at least 1 (36 ± 21), 2 (19 ± 12), 3 (10 ± 7), and 4 (6 ± 5) min under caffeine were associated with PAEE, [placebo: β=13, p=0.008 (1-min); β=20, p=0.006 (2-min); β=33, p=0.007 (3-min); β=40, p=0.009 (4-min); caffeine: β=10, p=0.006 (1-min); β=16, p=0.011 (2-min); β=23, p=0.033 (3-min); β=40, p=0.007 (4-min)]. A single bout of at least 1, 2, 3, and 4-min of MVPA resulted in an additional 13, 20, 33, and 40 kcal bout^-1^ in PAEE under placebo, and 10, 16, 23, and 40 kcal bout^-1^ in PAEE in caffeine condition. These associations remained significant when adjusted for sedentary time (<100 cpm).

## Discussion

The present investigation examined the effect of a moderate dose of caffeine on PAEE and the frequency of short bouts of both LIPA and MVPA, on real life settings and using objective methods such as DLW and accelerometry, respectively.

It is well known that caffeine works as an ergogenic substance for the central nervous system [10], then it would be expected an increase on daily PA, specifically the daily low intensity activities [40]. Unexpectedly, our novel finding revealed that a moderate dose of caffeine ingestion, corresponding to approximately 5 espresso cups of coffee (30 mL each) or 7 servings of tea, with each containing nearly 75 mg of caffeine had no effect on PAEE, the time spent in LIPA and MVPA, and the daily number of short bouts of both LIPA and MVPA intensities.

Previous studies indicated wide intra-individual variation in sedentary behavior and physical activity making it necessary to pay close attention to results *within* studies rather than comparing results between studies. Also, accelerometers in theory fail to record activity counts during certain very low intensity activities (LIPA) and thereby may underestimate the time spent in some activities [41–43]. We used a reliable type of accelerometer frequently used in the literature. Based on accelerometry data our participants performed approximately 2.5 hours of LIPA which is somewhat below the typical values presented for the Portuguese male population on this age range (3.3 hours) [11]. Furthermore, sedentary time (700 min) was also higher than the reference value (594 min). The high PAEE and other modest step count and MVPA suggest, that a large amount of energy expenditure occurs in LIPA and therefore the lack of change in PAEE (from DLW) confirms our findings that caffeine did not reduce the high amount of sedentary time in our participants because a shift toward more LIPA.

There is also some confusion concerning the best cutoff value to distinguish sedentary behavior and LIPA. The most commonly used is the 100 counts min^-1^, however based on a previous investigation it seems that this value is more accurate only when sedentary time is lower, while the 150 count min^-1^ is more accurate when sedentary time is higher. Despite participants being active, they also spent a major part of their waking day in sedentary behavior, therefore it could have been used the 150 cutoff value [44]. Two studies investigated the effects of caffeine on TEE in a metabolic chamber during 1-day [5,9]. Berube-Parent et al found an increase of 179 kcal in TEE considering a 600 mg day^-1^ caffeine dosage whereas the later investigation, using a lower caffeine dose (150 mg day^-1^) did not present significant differences between caffeine and placebo [9]. Though our mean caffeine dose administration was considerable higher than that reported by Dulloo et al [9], we also found non-significant effects of caffeine on TEE, over a 4-day period. Accordingly, the most likely explanation for these findings is probably related to the mean dosage used (~360 mg day^-1^), which is below the threshold for stimulating thermogenesis, i.e., 600–1000 mg caffeine day^-1^ [6]. In addition, a possible tolerance to caffeine effects [45] during the 4-day period may also explain these results. A previous investigation found significant effects of caffeine on increasing REE [3,46], even when participants were moderate caffeine consumers [4,47]. Although no study considered a period longer than 24 h, we were expecting that our low-caffeine users (<100 mg day^-1^) would increase REE under a 4-day caffeine intake. However, the results from the present investigation showed no significant increase in daily REE (13.6 kcal day^-1^; p=0.689).

It has been speculated that the contradictory results for all the EE components might be explained by the great variability on the individual response to caffeine due to body composition and dietary intake variables [48]. However, our findings did not confirm that fat-free mass or dietary intake mediated the effect of caffeine on PAEE, TEE, REE, and the frequencies of short bouts of both LIPA and MVPA.

There is still some uncertainty regarding the role of caffeine intake on both sedentary behavior and interruptions of sedentary time. Caffeine intake has been associated with lower sleep time and higher screen time, which are major sedentary behavior promoters [49]. Another investigation found small but statistically significant differences in spontaneous PA between catechins/caffeine treatment compared with the caffeine-only treatment, but no differences between caffeine-only and placebo conditions [50].

Recent research has focused on the detrimental effects of a sedentary lifestyle [51–53]. Researchers have hypothesized that breaking up sedentary behavior with LIPA and MVPA short bouts will improve the health of sedentary individuals, independently of PA levels [21]. However, to our knowledge only one study [23] investigated the acute contribution of PA short bouts of LIPA and MVPA to PAEE using direct calorimetry in laboratorial settings. We also found no significant contribution of short bouts of LIPA lasting less than 4-min to PAEE, which is also in agreement with previous findings [18].

The compendium of physical activities [54] documented “walking when gathering things at work ready to leave” has a metabolic equivalent of 3.0 METS or 10.5 ml kg^-1^ min^-1^, “walking less than 3.2 km on a firm surface” has 2.0 METS or 7.0 ml kg^-1^ min^-1^. Accordingly [23], observed that short bouts, with an intensity of 5.0 ml kg^-1^ min^-1^ were in the LIPA spectrum. This level of EE may possibly have an important impact on weight maintenance, or even possibly weight loss. Experimental studies should be conducted to test the effects of increasing PA short bouts on weight loss or maintenance.

It is important to mention some limitations and strengths of the current investigation. Our participants were already physically active at baseline which may have hindered or reduced the potentiating effect of caffeine on increasing PA levels. However, considering their low level of caffeine habituation we were not expecting increases in MVPA but in spontaneous LIPA. Still, these participants had about 700 min/day of sedentary time as measured with our accelerometers and thus ample time for caffeine to cause individuals to “get up and move” if caffeine were to stimulate more LIPA or some other kind of activity.

Hip-worn accelerometers have been used to estimate sedentary time from total body movement. While accelerometers function well for many purposes, most models are not designed to accurately measure postures like sitting and standing. Therefore, as presented earlier in this section, the use of an accelerometer to measure sedentary time and LIPA transitions may be less sensitive and somehow underestimated LIPA.

We did not assess hormonal changes that, in fact, could vary under caffeine intake, which would likely affect EE, specifically during rest. Studies indicated that caffeine increases catecholamine production [9,48,55] however, we did not find significant differences between REE values assessed at baseline and at the end of each condition. Our findings are only generalized to non-obese physically active males, low-caffeine users, and for a 4-day period. Further research should be conducted with a higher caffeine dose and in a population that vary in age, body mass index, gender, and activity levels. The major strengths of this study include the study design with double-blind randomized crossover trial and state-of-the-art methods for assessing PAEE, specifically the DLW technique for TEE in combination with indirect calorimetry for REE in free living conditions.

In conclusion, our findings revealed that resting, total, and physical activity EE, and accelerometry measurements of both LIPA and MVPA were not affected by the ingestion of a moderate dose of caffeine. Also, a significant contribution to PAEE was observed in males that increased short bouts frequency of at least 1 up to 4-min of MVPA and at least 4-min of LIPA. Regardless of sedentary time, shifting sedentary behavior to LIPA through bouts of at least 4-min can markedly increase PAEE in healthy males.

## Supporting Information

Checklist S1 CONSORT Checklist. (DOC)

Protocol S1 Trial protocol. (PDF)
