# Supplementary material for: Caffeine Intake, Short Bouts of Physical Activity, and Energy Expenditure: A Double-Blind Randomized Crossover Trial
Source: PLoS One. 2013 Jul 15;8(7):e68936. doi: 10.1371/journal.pone.0068936 (PMC3711911; doi:10.1371/journal.pone.0068936)
Supplement: Protocol S1 — (PDF) [file pone.0068936.s002.pdf]

ClinicalTrials.gov
Protocol Registration System

Protocol Registration Receipt
11/18/2011

Effects of Caffeine in Hydration and Energy Metabolism

This study has been completed.

Sponsor:	Technical University of Lisbon
Collaborators:	
Information provided by (Responsible Party):	Analiza M Silva, PhD, Technical University of Lisbon
ClinicalTrials.gov Identifier:	

Purpose

The main purposes of this randomized cross-over trial are to characterize and compare the effects of a moderate dose of caffeine intake in healthy physically active males on hydration and energy metabolism specifically:

a. Total body water and its compartments (intracellular and extracellular) assessed by by dilution techniques, and hydration state by urine specific gravity;
b. Total energy expenditure by double labeled water, resting energy expenditure by indirect calorimetry, and physical activity energy expenditure;
c. Energy expenditure in physical activity of daily living, in particular duration and intensity of activity.
d. If the changes occurred between placebo and caffeine ingestion on the main outcomes were dependent on specific covariates, namely body composition and dietary intake.

Condition	Intervention	Phase
Caffeine	Drug: Caffeine<br>Drug: Placebo	N/A

Study Type: Interventional
Study Design: Prevention, Crossover Assignment, Double Blind (Subject, Investigator), Randomized, Efficacy Study

Official Title: Effects of Caffeine Ingestion in Total-body Water, Extra and Intracellular Water Distribution, and Energy Metabolism

Further study details as provided by Analiza M Silva, PhD, Technical University of Lisbon:

Primary Outcome Measure:

- Changes in Total body water (TBW), extracellular water (ECW), intracellular water (ICW), and hydration status from baseline to each experimental condition (Placebo or Caffeine) [Time Frame: day 0, day 4, and day 11] [Designated as safety issue: No]

  TBW was assessed by deuterium dilution using a stable Hydra gas isotope ratio mass spectrometer (PDZ, Europa Scientific, Crewe, United Kingdom).ECW was assessed by the sodium bromide dilution method. ICW was calculated as the difference between TBW and ECW. Hydration status was determined based on urine specific gravity (USG) measured by a refractrometer (Urisys 1100 Urine Analyzer, Roche, Portugal)

- Changes in total energy expenditure (TEE), resting energy expenditure (REE), and physical activity energy expenditure (PAEE) between each experimental conditions (placebo or caffeine) [Time Frame: Day 4 and day 11] [Designated as safety issue: No]

  TEE was assessed by the double labeled water technique, REE by indirect calorimetry, and PAEE calculated as PAEE= TEE - (0.1xTEE + REE), assuming that 10% of TEE is due to the thermogenic effect of food

- Changes in daily time spent in sedentary activities (DTSS), light activities (DTSL), moderate activities (DTSM), and vigorous activities (DTSV) between each experimental conditions (placebo or caffeine) [Time Frame: day 4 and day 11] [Designated as safety issue: No]

  DTSS, DTSL, DTSM, and DTCV were assessed by accelerometry using cut-off values based on specific counts/min interval

Secondary Outcome Measures:

- Changes in body composition from baseline to each condition (Placebo or Caffeine) [Time Frame: day 0, day 4, and day 11] [Designated as safety issue: No]

  Body composition was assessed by dual energy x-ray absorptiometry (DXA)

- Changes in dietary intake (energy and macro nutrient) between each condition (placebo and caffeine) [Time Frame: Day 4 and day 11] [Designated as safety issue: No]

  Dietary intake was assessed using a 24-h dietary records.

Enrollment: 30
Study Start Date: January 2010
Study Completion Date: March 2010
Primary Completion Date: March 2010

Arms	Assigned Interventions
Experimental: Caffeine<br>Intervention with Caffeine in a random order	Drug: Caffeine<br>5 mg/ kg of body weight (capsules), twice a day (morning and afternoon), during 4 days.

Arms	Assigned Interventions
Placebo Comparator: Placebo<br>Placebo (malt dextrin) administered in a random order	Drug: Placebo<br>Malt dextrin (5 mg/kg body mass) twice a day during 4 days

Although the effect of caffeine on hydration status, usually assessed by a urine specific gravity test (USG) has been extensively studied no research has been conducted to analyze its effects on total body water and its compartments using reference methods. In addition some uncertainty still remains about the influence of caffeine ingestion on hydration due to methodological limitations, specifically the use of less valid techniques for total-body water assessment, small sample size, and the lack of control for potential confounding factors. The information above specifically the methodological gaps mentioned and an inadequate experimental design lead us to further understand the effect of a moderate dose of caffeine in total body water (TBW) and its intra (ICW) and extracellular (ECW) compartments in non-caffeine consumers during a short-term period (4 days). Additionally, though caffeine effects on energy expenditure has been studied, its influence on physical activity (PA) during free-living conditions using gold standard and objective measures of PA is limited. Therefore, we also investigated the impact of a moderate dose of caffeine on resting energy expenditure (REE), PA energy expenditure (PAEE), total energy expenditure (TEE), and daily time spent in sedentary (DTSS), light (DTSL), moderate (DTSM), and vigorous (DTSV) intensity activities in non-obese physically active males. To overcome the methodological gaps in previous studies we assessed dietary intake and objective measures of physical activity throughout the experimental trial to assure that the same diet and physical activity patterns were maintained. Also it was analyzed if the effect of caffeine was independent of body composition, assessed by dual-energy x-ray absorptiometry.

To perform this research study, a total of 30 non-smoker males, low caffeine users (<100 mg/day), aged 20-39 yrs [body mass (BM): 72.7 ± 8.8 kg; Height: 1.77 ± 0.07 m] were followed in a double-blind crossover experimental design with two conditions in a random sequence: caffeine (5 mg per kg of BM/day) and malt-dextrine as placebo, both through capsules. Conditions lasted for 4 days with a 3-day washout period.

Evaluations were performed at baseline (visit-1), end of condition 1 (visit-2), and end of condition 2 (visit-3). Fat and fat-free masses (FFM) were assessed by dual energy x-ray absorptiometry. TBW and ECW were determined by dilution techniques (deuterium and bromide, specifically) while ICW was calculated as the difference between TBW and ECW (Schoeller et al., 1980. Total body water measurement in humans with 18O and 2H labeled water. Am J Clin Nutr 33:2686-2693). TEE was assessed using doubly labeled water technique by administrating two stable isotopes 2H (deuterium) and 18O (oxygen 18) with a respective dose of 0.1 g / kg and 1.8 g / kg of body water (Schoeller DA, van Santen E, 1982. Measurement of energy expenditure in humans by doubly labeled water method. J Appl Physiol 53:955-959). REE was assessed by indirect calorimetry and PAEE calculated as [TEE-(REE+0.1TEE)]. An accelerometer (ActiGraph, GT1M model, Fort Walton Beach, Florida) was used to estimate DTSS, DTSL, DTSM, and DTSV. The cutoff values used to define the intensity of physical activity and therefore to quantify the mean time in each intensity (sedentary, light, moderate or vigorous) for persons aged 18 years or older were: sedentary: < 100 counts/min; light: 100-2019 counts/min; moderate: 2,020-5,998 counts/min (corresponding to 3-5.9 METs); vigorous: ≥ 5999 counts/min (corresponding to ≥ 6 METs) (Troiano et al. Physical activity in the United States measured by accelerometer. Med Sci Sports Exerc. 2008;40:181-8).

Eligibility

Ages Eligible for Study: 18 Years to 39 Years
Genders Eligible for Study: Male
Accepts healthy volunteers.

Inclusion Criteria:

- male
- aged 18-39 years old
- body mass index between 18.5 and 29.9 kg/m2
- Subjects had to be free of any major disease with a general healthy status warranted

Exclusion Criteria:

- Smokers
- taking medication or dietary supplements that may interfere with hydration status
- body composition or energy expenditure regulation
- Caffeine consumption > 100 mg/day

Contacts and Locations

Locations

Portugal

Faculty of Human Kinetics
Lisbon, Portugal, 1495-688

Investigators

Principal Investigator:	Analiza M Silva, PhD	Faculty of Human Kinetics, Technical University of Lisbon

More Information

Institute of Hydration and Health
http://www.ihs.pt

Results Publications:

Silva AM, Armada-da-Silva P, Sardinha LB, Júdice PB, Magalhães JP, Matias CN & Santos DA. Efeitos da ingestão de cafeína na água corporal total, distribuição de fluidos intra e extracelulares e no dispêndio energético. A Report for the Institution of Hydration and Health, In press.

Júdice PB (2011). Determinant Factors on hydration status assessed by Urine Specific Gravity: accuracy of technical procedures and effect of caffeine ingestion. Master's Degree Thesis. Faculty of Human Kinetics-Technical University of Lisbon, Cruz-Quebrada.

Magalhães JP, Santos DA, Silva AM, Matias CN, Júdice PB, Quintas IC, Sardinha LB (2011). Total energy expenditure: combined heart rate and motion sensor vs uni-axial accelerometry models Book of abstracts of the International conference on Recent Advances and Controversies in Measuring Energy Metabolism, pp

106.

Quintas IC, Santos DA, Matias CN, Silva AM, Gobbo, LA, Gonçalves EM, Magalhães JP, Júdice PB, Sardinha LB (2011). Does caffeine intake affect resting energy expenditure? Book of abstracts of the International conference on Recent Advances and Controversies in Measuring Energy Metabolism, pp 104.

Júdice PB, Silva AM, Magalhães JP, Matias CN, Santos DA, Armada-da-Silva P, & Sardinha LB. Does caffeine intake increases energy expenditure and habitual physical activity? A double-blind randomized crossover trial. Medicine and Science in Sports and Exercise. In Review.

Responsible Party:	Analiza M Silva, PhD, Principal Investigator, Technical University of Lisbon
Study ID Numbers:	IHS-2010
Health Authority:	Portugal: National Pharmacy and Medicines Institute
